# Influence of Exposure Period and Angle Alteration on the Flexural Resilience and Mechanical Attributes of Photosensitive Resin

**DOI:** 10.3390/nano12152566

**Published:** 2022-07-26

**Authors:** Sadaf Bashir Khan, Nan Li, Jiahua Liang, Chuang Xiao, Xiaohong Sun, Shenggui Chen

**Affiliations:** 1 Dongguan University of Technology, Dongguan 523808, China; sadafbashirkhan@dgut.edu.cn (S.B.K.); dglgln@163.com (N.L.); xiaoxcuang@163.com (C.X.); 2 School of Art and Design, Guangzhou Panyu Polytechnic, Guangzhou 511483, China; 3 School of Materials Science and Engineering, Tianjin University, Tianjin 300350, China; 4 Dongguan Institute of Science and Technology Innovation, Dongguan University of Technology, Dongguan 523808, China; jiahua_liang1114@163.com

**Keywords:** acrylic resins, physical properties, strength, exposure time, flexural durability, stiffness, photosensitive resin

## Abstract

Despite the large number of studies addressing the effect of acrylic resin polymerization concerning flexural properties, limited research has been conducted on the manufacturing impact on a polymer’s mechanical properties. Photosensitive resinous materials are used in various engineering applications where they may be exposed to multiple detrimental environments during their lifetime. Therefore, there is a need to understand the impact of an environment on the service life of resins. Thus, flexural tests were conducted to study the effects of exposure time and angle on the flexural strength of resins. Herein, the main objective was to explore the strength, stability, and flexural durability of photosensitive resin (EPIC-2000ST) fabricated at different exposure times (E) and angle deviation varying from 0° to 85° with a 5° increment. The samples in circular rings were manufactured and divided into five groups according to their exposure time (E): 10 s, 20 s, 30 s, 40 s, and 50 s. In each exposure time, we designed rings via SolidWorks software and experimentally fabricated at different oblique angles (OA) varying from 0° to 85° with a 5° increment during each fabrication, i.e., OA = 0°, 5°, 10°, 15°, 20°, 25°, 30°, 35°, 40°, 45°, 50°, 55°, 60°, 65°, 70°, 75°, 80°, and 85°. Flexural strength was evaluated using a three-point bending test. Optical electron microscopy was used to examines the samples’ exterior, interior, and ruptured surfaces. Our experimental analysis shows that flexural strength was significantly enhanced by increasing exposure time and at higher oblique angles. However, at lower angles and less exposure time, mechanical flexural resilience declines.

## 1. Introduction

Due to increasing worldwide development and difficulties in employing metallic materials in industrial tribological applications, the tribological behavior of polymer composites has seen inventive growth and the attention of many researchers [1]. Fiber-strengthened polymeric composites have been developed with considerable advantages over metallic substances due to mechanical features that lead them to practical applications [2]. Different research teams have been interested in the tribological features of resin materials [3]. Several studies on resin application in brakes, clutches, bolts, and nuts have found that friction and wear efficiency are essential [4,5]. Wear, or the resistance of a solid surface to be removed, has been defined in terms of weight loss, wear resistance, and specific wear rate [6]. It is popular to use epoxy as a bonding agent since it does not shrink after curing and may be utilized at high temperatures [6]. 

A growing number of researchers are interested in learning more about epoxy’s mechanisms due to the numerous advantages it offers. The qualities of a photosensitive resin can be altered by various factors, including heat, stress, moisture, corrosive compounds, or exposure to ultraviolet rays [7]. Photosensitive resin (PSR) elasticity and durability evaluations have been subjected to a wide range of studies [8]. The dynamics of fluids and aqueous solutions on acrylic-reinforced epoxy composites have indeed been thoroughly examined [9]. Water has long been known to affect the mechanical characteristics of PSR and composites significantly. One can alter the properties of PSR and reinforcing fibers by combining aqueous solutions with varying pH values, although the fabrication and impact of exposure time are not comprehensively investigated [10]. The cracks in the matrix and how defects interact with the matrix are not fully comprehended [1]. One cannot express how a material will break just on its tensile strength or modulus. Exactly what measurable features are relevant to its real-world behavior have yet to be determined.

In the case of 3D fabrication, stereolithography apparatus (SLA), digital light processing (DLP), fused deposition modeling (FDM), and powder bed fusion (PBF) are some of the most commonly used 3D printing technologies [11,12]. To create a 3D object that has the appearance of depth, they employ a variety of materials and construction techniques. SLA and DLP are the two most common forms of 3D printing utilized in engineering applications [13,14]. In this methodology, a photosensitive liquid resin bath is used to contain the liquid resin, and an ultraviolet (UV) light is used to cure the resin to accelerate the process. SLA develops solid parts in a model-making platform by building them up layer by layer. In 1986, it was employed for 3D systems for the first time [15]. Multiple layers are applied and cured to create a long-lasting product. DLP 3D printing uses a digital light projector instead of a laser to cure the photopolymer resin, but the technique and technology are identical [8]. DLP 3D printing offers a significant speed advantage compared to normal SLA because it can print and cure an individual stratum throughout the entire platform in only a few seconds [8]. Making objects with DLP is also less expensive than manufacturing them with SLA or other 3D printing technologies. Models from digital impressions, including surgical guides, castable restorations, and even temporary crowns, can now be created using DLP printing. DLP printing is expected to rise as a result of its speed and accuracy [5,10].

The accuracy and reliability of 3D printing have improved significantly in recent years, making it an excellent option for the biomedical and automobile industry [14]. Medical and dental sciences, orthopedics, bioengineered tissues, and medical devices have seen new uses for 3D printing [10,16]. By creating an STL (standard triangle language) file and then 3D printing tiny volume sections together, one may quickly turn 3D models into tangible items [17,18]. The exploration of materials has advanced in recent decades, following significant and notable progress associated with material aspects and technology and fundamentally altering dental materials using restorative approaches [4]. For decades, practitioners have struggled to find biocompatible materials for prosthetics and restorations and to achieve natural-appearing prosthetics and restorations that resist harsh oral environments [3,19]. As soon as PSR was introduced to the denture-base manufacturing industry, it quickly became a popular material of choice [20]. Even though dental implants are increasingly being utilized to replace missing teeth in partial dentate and edentulous patients, PSR are still the material of denture bases due to their strength and flexibility [21,22]. Due to a variety of variables, bisphenol A dimethacrylate has grown in popularity. These include its ease of manipulation and processing, equipment affordability, and aesthetic appeal. Fracture is a considerable danger because traditional denture bases are prone to mechanical failure, making them brittle [17,22].

Adjusting a number of variables can boost the dependability of 3D-printed products, such as precision, reliability, durability, scan speed, film thickness, and curing procedures. The environment can have an impact on architecture and restorative PSR materials [23,24]. Chemical and temperature changes in the environment can affect the material properties, making it particularly difficult to work in. Water can alter the mechanical characteristics of composite based PSR [14,25,26]. Material that has been kept wet makes it more difficult to bend when making an occlusal device. The mechanical qualities of dental restorations, such as fracture resistance and flexural strength, might be adversely affected by artificial aging procedures and the polymerization process [2]. Research on PSR materials and 3D printing direction flexibility and strength identified a correlation between resin-based materials and their 3D printing flexibility [27].

In numerous studies, researchers have looked at how 3D-printed materials operate on the surface and how well they work. Flexural strength, surface roughness, hardness, and aesthetics have all been considered [28,29]. Adequate research on the effects of manufacturing and postprinting circumstances on the mechanical and physical characteristics of 3D-printed materials is required [30]. It is important to know how different factors affect the printed materials’ mechanical attributes to improve the quality of restorations and their performance in everyday use. Acrylic PSR polymerization and its flexural properties have been the subject of numerous studies [31,32,33]. There has been minimal investigation into how manufacturing alters PSR mechanical properties [34]. They can be subjected to many harmful things during their lifetime in various engineering applications. It is essential to understand how environmental factors influence PSR life expectancy. As a result, herein, we fabricated PSR rings and determined how exposure time and angle affect their mechanical strength.

## 2. Materials and Methods

We used a 3D printer (Moon Ray) to fabricate PSR ring specimens in our experiments. The PSR used in the manufacturing process is named EPIC-2000ST (batch number: 20220321E2T), purchased from Shenzhen Yongchanghe Technology Co., Ltd. (Shenzhen, China). We used this PSR because of its easy casting, low odor, and volume-shrinkage properties. Table 1 represents the impact of exposure time and the time required to complete the fabrication process of circular rings. Our experimental results determine two important aspects. Higher oblique angles (OA) require more time to complete the fabrication process of rings than lower oblique angles. Secondly, increasing exposure time (E) increases the time needed for ring formation. During the fabrication process, we kept all other parameters, i.e., support density, including base height (2.3 mm), support spacing (6.1 mm), upper contact size (0.55 mm), and support height (4.5 mm), consistent. The slice exposure time was a second per slice.

### 3D Printed PSR Specimen Fabrication

**Scheme 1:**
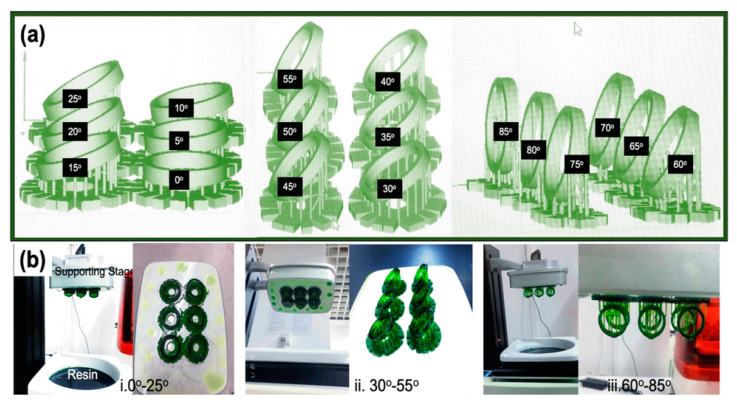
Dimensional model of fabricated rings at different oblique angles, (**a**) 3D Model designs (**b**) experimentally 3D printed rings.

The circular ring specimens were first designed (SolidWorks software) and saved as a standard tessellation language (STL) file, as shown in Scheme 1. The STL file was used to create the 3D-printed specimens, which were then constructed and encoded into a 3D printer. After initial manufacturing, the samples were thoroughly washed in an isopropyl alcohol bath per the relevant requirements. Ten samples at each exposure time and angle were fabricated using the same methodology. Hence, 180 specimens were fabricated at each exposure time, and 1000 samples were prepared and analyzed for this study. The average results are reported here.

## 3. Results

**Figure 1:**
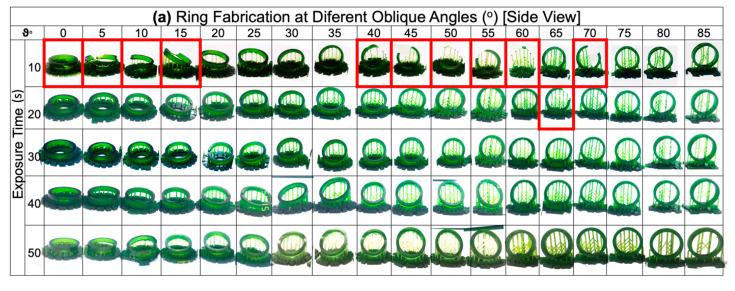

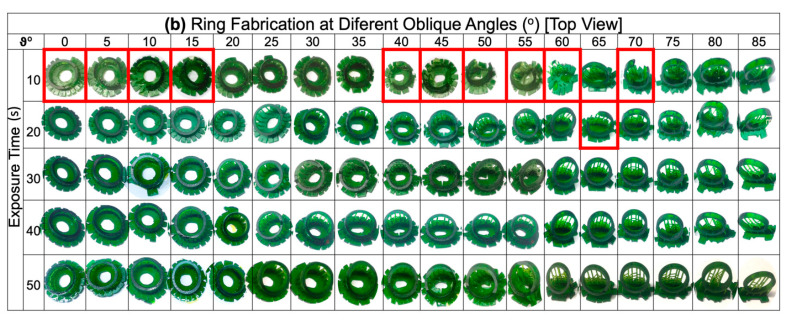
(**a**) Side and (**b**) top views of fabricated rings at oblique angles formulated at a different exposure time.

We selected E = 10 s and fabricated rings at different oblique angles varying from 0° to 85°. Our experimental results demonstrated that the incomplete formation of rings appeared at 0°, 5°,10°, 30°, 35°, 40°, 60°, and 70°, as shown in Figure 1a,b. The rings were imperfect, partially finished, having some flaws at edges, defective/damaged, or had cracks, as shown in Figure 1a,b, displaying side and top views of fabricated rings. We repeated our experiments nearly ten times and got almost the same results. However, we got a complete ring formed with certain surface imperfections once or twice, including distorted, one-sided slanted, or slightly cracked surfaces. We changed the orientation and position of rings in the software during the fabrication process in the support stage, but the formation of fully complete rings was seldom achieved. 

We used fresh and new PSR, but got almost the same results, i.e., half-finished ring formation occurred at E = 10 s. When we increased the E to 20 s, ring formation eventually occurred at all oblique angles. However, at 65°, ring formation did not occur, even after several experimental repetitions. An incomplete, partial ring formation occurred, as visualized in Figure 1a,b, displaying side and top views. A smooth, well-finished, and homogeneous circular ring developed with increasing exposure time from E = 30 s to 50 s (Figure 1a,b).Thus, the ring structure’s configuration, attributes, and accuracy were enhanced with exposure time. A gradual rise in consistency and evenness in ring structure was observed with increasing the exposure time.

### 3.1. 3D Printed PSR Structural Analysis

**Scheme 2:**
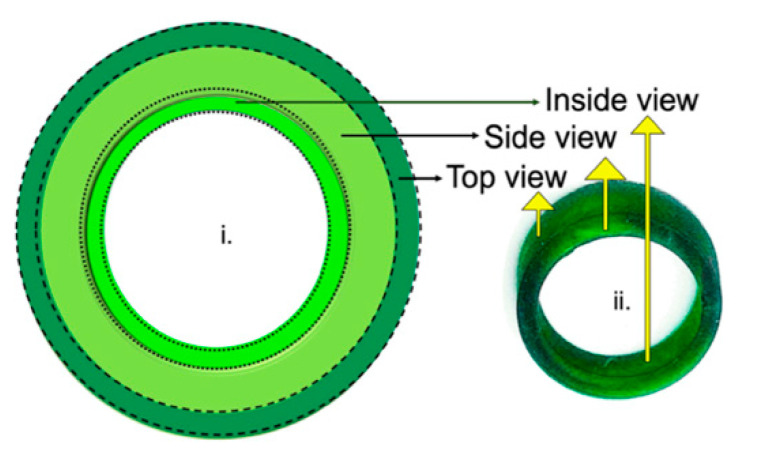
Images indicating the point of analysis of fabricated ring (**i**) model design (**ii**) 3D printed ring.

**Figure 2:**
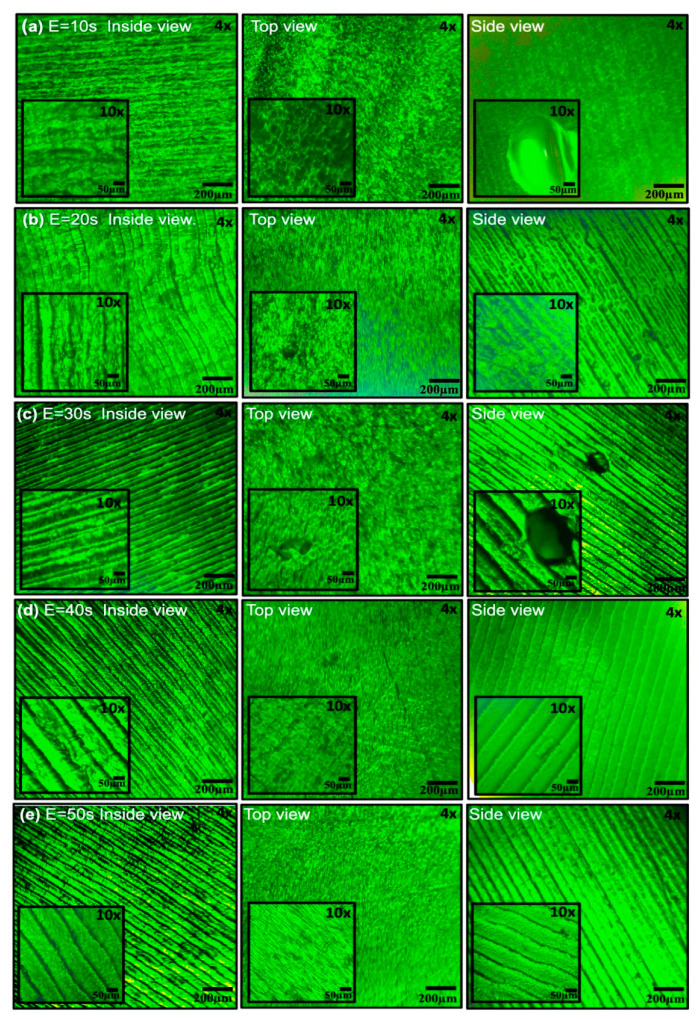
Optical images of ring surface at (**a**) E = 10 s, (**b**) E = 20 s, (**c**) E = 30 s, (**d**) E = 40 s, and (**e**) E = 50 s. The inset in all images represents the magnified image of the same sample.

To evaluate the steady upsurge in the uniformity and symmetry of ring texture at different oblique angles, we analyzed the ring surface using an optical microscope, as shown in Figure 2. We examined the surface of the ring from three different positions (inside, top, and, side surface analysis) as specified in Scheme 2*,* illustrating (i & ii) the point of analysis via graphical representation of the ring and fabricated ring. The textures of the surfaces fabricated at different exposure times were investigated using an optical microscope. The results are displayed in Figure 2.

All samples examined at three distinctive positions exhibited slightly varying surface textures. The inside and side views demonstrated the stratum morphology, while the top view displayed a dense porous structure. At E = 10 s, the inside and side views depicted an imperfect partial formation of surface layers. The surface layers were highly nonuniform and inhomogeneous, including many cavities, flaws, and the presence of numerous sizes of vacuities. However, at E = 20 s, a slight appearance of layer development seemed to occur, but still, it showed nonuniform texture, with some empty spots, presence of voids, and nonadjacent layers. In the case of E = 30 s, one could visualize the occurrence of homogeneous layers adjacent to each other with fewer defects, having borderlines. 

However, at E = 40 s and E = 50 s, the surface texture became more developed with proper edges. The layers were highly oriented, homogeneous, well organized, and adjacent to each other with negligible imperfection and minor flaws (i.e., cavities still found at E = 40 s; side view). The top-view ring-surface texture displayed a highly porous and spongy assembly, which declined gradually with increasing exposure time from 10 s to 30 s. A few cracks and voids were observed from the top view at E = 20 s and E = 30 s. However, uniform and consistent thin film configuration was observed at E = 40 s and E = 50 s. Thus, the optical images validate that the surface of the ring became more developed, with improve attributes and quality with increasing exposure time, as shown in Figure 2.

**Figure 3:**
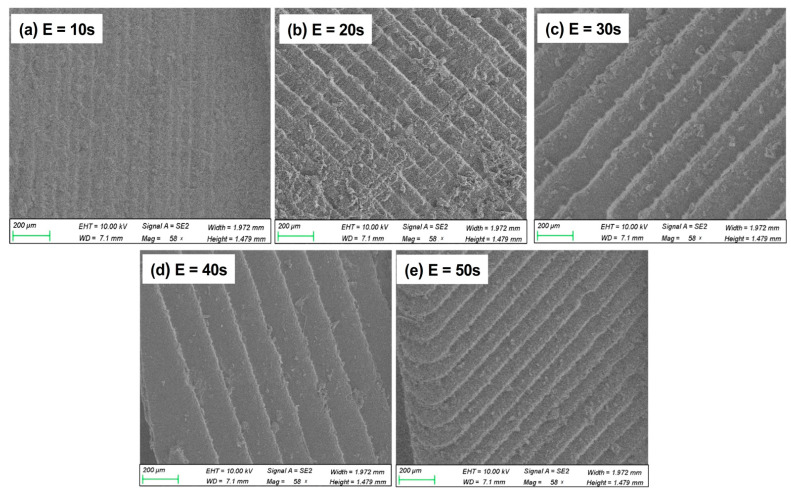
SEM images of the fabricated ring at (**a**) E = 10 s, (**b**) E = 20 s, (**c**) E = 30 s, (**d**) E = 40 s, and (**e**) E = 50 s represent the structural analysis.

Figure 3 displays SEM images demonstrating the morphology and apparent consistency at different exposure times. The structure was more refined at a higher exposure time and had sharper edges (E = 30 s, 40 s, and 50 s). All the layers were strongly aligned, homogenized, and next to one other with small imperfections and faults. At E = 10 s and 20 s, the ring-surface texture is in the initial developing phase with the formation of strata. At E = 10 s and E = 20 s, a few cracks and voids were spotted from the top perspective. However, at E = 40 s and E = 50 s, a homogeneous and constant thin film structure was found. As illustrated in Figure 3, the SEM images reveal that the ring’s surface became more advanced with more features and improved attributes as exposure time increased.

### 3.2. 3D Printed PSR Flexural Testing

**Scheme 3:**
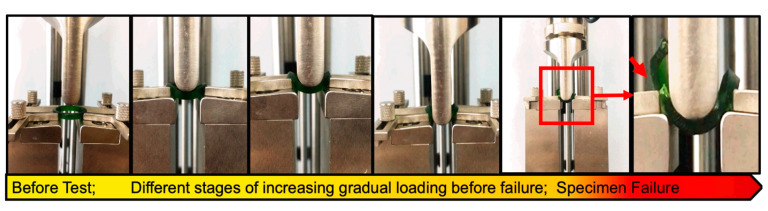
Indicates the fracture point of failure analysis of fabricated ring in 3-point bending test.

The flexural strength test was performed as per ISO 1567:1999. The configuration, including the thickness, height, and width of each ring, was measured using a digital Vernier caliper (Aerospace, Dongguan, China), measuring at an accuracy of ±0.1 mm before performing the flexural strength testing. The mechanical strength tests of the specimens were conducted via a microcomputer-controlled electronic universal testing machine (LD23.104, LSI). The test was performed using the three-point technique with a test span of 15 mm and a beam-travel speed of 0.5 mm/min. The ring was placed and fixed in the middle of the supporting stage (Scheme 3). The loading wedge was set to travel at a cross-heading speed of 0.5 mm/min, focusing on the center of the upper exterior of the circular ring. The rings were loaded gradually and consistently until failure occurred. An infographic recording device kept track of the peak load (fracture load). Before fracture (F), the maximum load was specified in Newtons on the display of the testing machine. Flexural strength (σ) was calculated:

σ = 3FL/(2BH^2^) (1)

Here, F is the maximum load (Newtons), L is the span length (millimeters), B is the width of the samples (millimeters), and H is the height (millimeters).

### 3.3. 3D Printed PSR Stress Analysis

**Figure 4:**
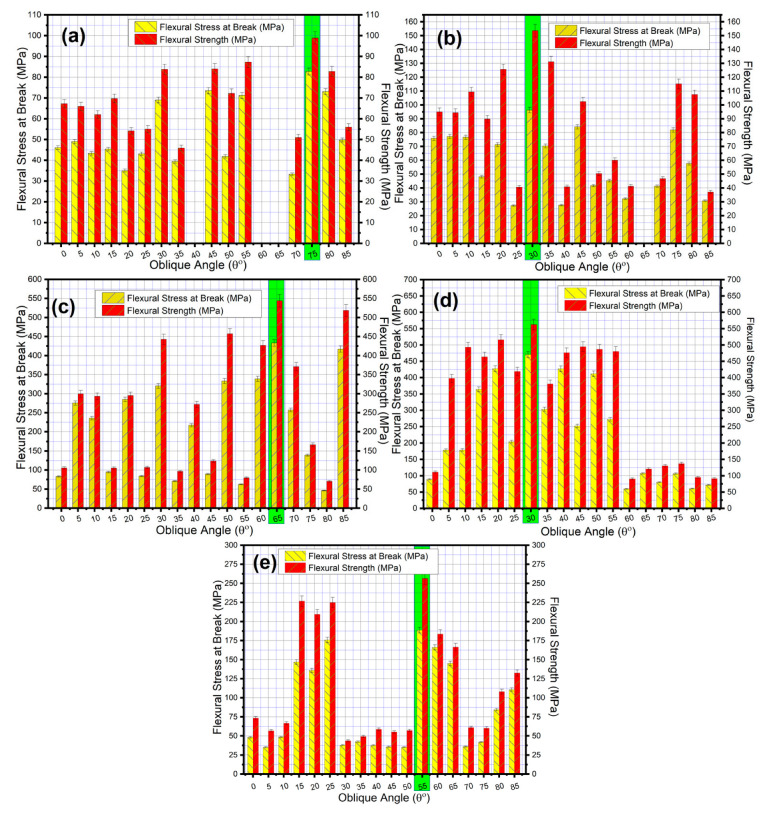
Results of a statistical analysis of flexural strength at (**a**) E = 10 s, (**b**) E = 20 s, (**c**) E = 30 s, (**d**) E = 40 s and (**e**) E = 50 s.

The statistical study’s experimental outcomes for flexural strength are displayed in the bar graph in Figure 4. At E = 10 s, maximum flexural strength was observed at OA of 75°. However, at lower OA (0° up to 15°), the rings showed mechanical strength of nearly >60 MPa < 70 MPa. In the case of the middle-region OA (25–65°), no ring fabrication took place at 40°, 60°, or 65°, even after many repetitions. There was no ring formation at all, so we could not proceed with the experiment. There was a consistency in flexural strength at OA 45°, 50°, and 55° having flexural strength >70 Mpa < 80 MPa. Thus, a specified trend of flexural strength at varying OA was observed, i.e., decreasing to increasing and declining at the highest angle. A similar phenomenon was observed in the case of flexural stress before the fracture occurred in rings at E = 10 s, as shown in bar graph Figure 4a.

In the case of E = 20 s, the maximum flexural strength was observed at an OA of 30° [>150 MPa]. At lower OA (0–20°), there was consistency and a gradual increment in mechanical strength from nearly >90 MPa to <125 MPa. Then, a sharp decrement was observed at OA 25° and a sudden increment at 30°. In the case of middle-region OA (35–60°), the flexural strength of the fabricated rings declined with an increasingly oblique angle from 137 MPa to 41 MPa. Again, no ring fabrication took place at 65°. At higher OA, there was still lower flexural strength, varying from 37 MPa up to 115 MPa. Thus, a trend of increasing to decreasing flexural strength at varying OA was observed. The rings underwent flexural stress before rupturing at E = 20 s, following a similar trend of increasing stress and decline in stress at higher OA. However, the rings experienced higher stress than E = 10 s at different OA, except at 85°. Besides this, the flexural strength of the rings was slightly improved by increasing the exposure time at different OA (E40 s > E30 s > E20 s = E50 s < E10 s).

In the case of E = 30 s, the maximum flexural strength was observed at OA of 65° [>544 MPa]. At lower OA (0–30°), there was a uniformity and a steady rise in mechanical strength from nearly >105 MPa to <442 MPa. In the case of middle-region OA (35–60°), a sharp decrement was observed at OA 35° and 55° and a sudden increment at 50°. Higher OA still showed lower flexural strength, varying from 371 MPa to 518 MPa. Thus, a cyclic trend of increasing decreases in flexural strength at varying OA was perceived herein. The rings underwent flexural stress before rupturing at E = 20 s, following a similar trend of increasing stress and decline in stress at higher OA. Moreover, the flexural strength of the rings was slightly improved with increased exposure time at different OA, e.g., E = 30 s at angle 10° (105 MPa) showed greater flexural strength than E = 10 s at an angle of 75° (98.9 MPa). Generally, at all angles, flexural strength increased at E = 30 s compared to E = 20 s and E = 10 s.

At E = 40 s, the rings showed the best flexural strength among all exposures. The maximum flexural strength was at an OA of 30° [>562 MPa]. A rising flexural strength trend was observed at lower and medium OA (0–55°). There was a uniformity and a steadiness in the mechanical strength varying from >397 MPa to <562 MPa and a declining inclination at higher OA (60–85°), showing consistency in flexural strength varying from 90 to 106 MPa. Besides this, the rings showed a similar trend in flexural stress. Before undergoing final damage, rupture occurred at 470 MPa at OA 30°. This followed an increase and a decreasing trend.

The bar graph shows that the flexural strength of rings increased with increasing exposure time. At E = 10 s, there was poor flexural strength compared to other exposure times due to the incomplete formation of rings. The specimens had certain imperfections, such as cavities, cracked edges, or slightly broken corners. Flexural strength improved gradually by increasing the exposure time, as observed at E = 20 s, due to a decline in voids and surface imperfections. Still, the ring showed good strength at E = 30 s, further enhanced at E = 40 s because of the increased degree of homogeneity and its appropriate structure, curvature, and ability to bend. However, with further increment in exposure time, the flexural strength declined at E = 50 s. There was less flexural strength than E = 30 s due to slightly firm assemblage aggregation, making it harder and brittleness to begin to occur, which reduced the flexural strength in rings. We also evaluated the 3D-printed PSR strain (ε_fB_ and ε_fM_), provided in Supporting Information (Figure S1).

### 3.4. 3D Printed PSR Load-Displacement Graph and Failure Patterns

**Figure 5:**
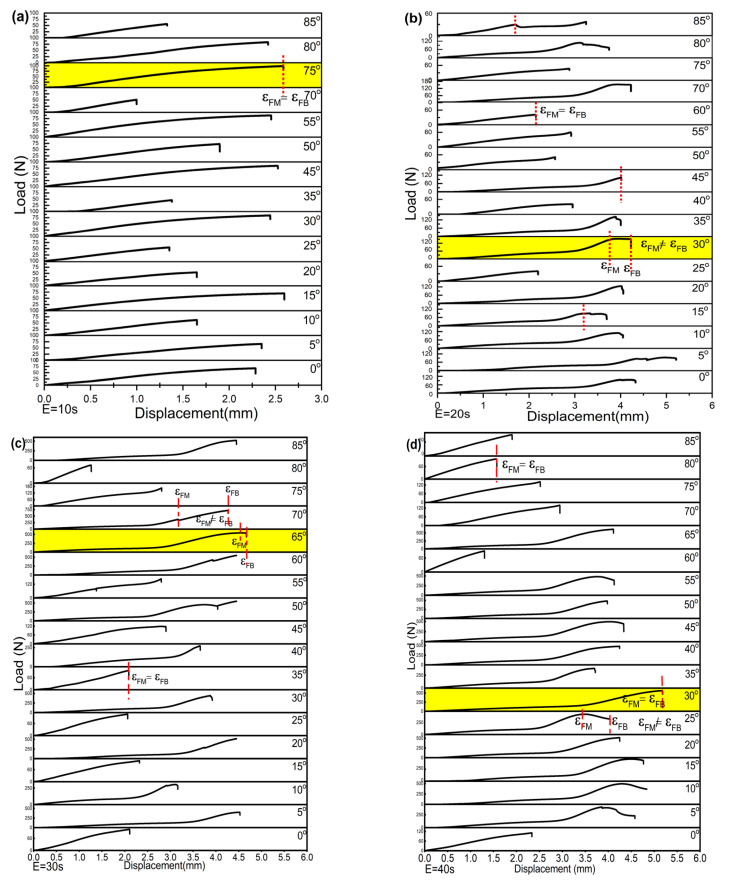

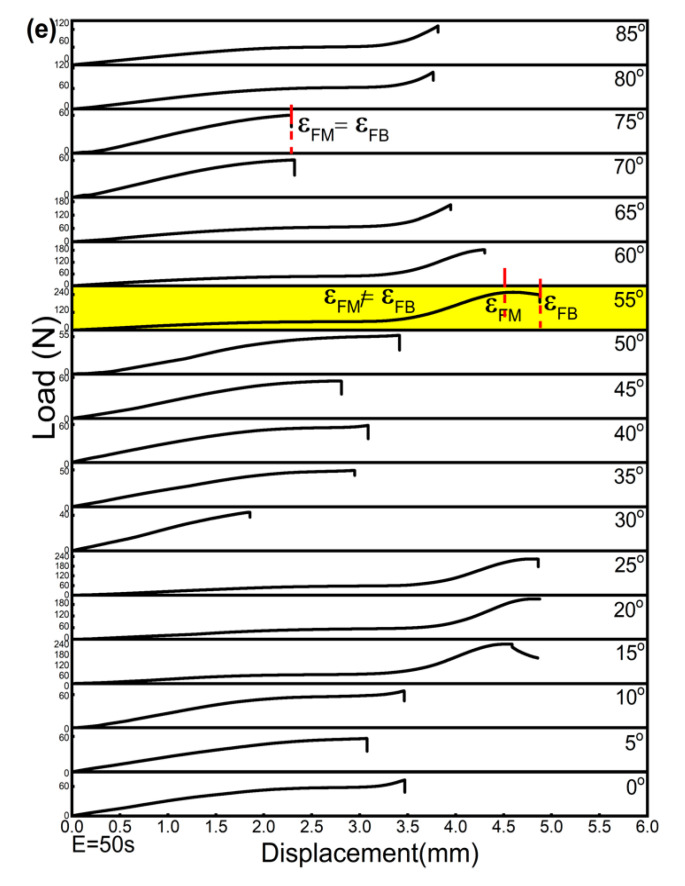
Typical load-displacement curves of the 3D-printed PSR rings at (**a**) E = 10 s, (**b**) E = 20 s, (**c**) E = 30 s, (**d**) E = 40 s, and (**e**) E = 50 s at different OA.

Numerous technical applications utilize PSR materials, which can be subjected to harsh environments during their service life. Understanding the influence of exposure time on PSR working life is critical. This is why flexural experiments on PSR were conducted to observe the influence of exposure and angle on PSR flexural strength. The rings slowly bent as the weight increased until failure occurred, and the test was terminated. Figure 5 shows the typical load-displacement curves of the 3D-printed PSR rings at various exposure times, exhibiting the amount of elongation at breaks as stated. After printing, all 3D-printed specimens exhibited linear elastic deformation before yielding a point at E = 10 s. Before breaking, the specimens showed signs of plastic deformation. Regardless of the angle at which they fractured, all samples demonstrated a brittle fracture mode at E = 10 (Figure 5a).

In the case of E = 20 s, all the load-displacement curves had an initially linear response, which became progressively nonlinear above displacements of 4 mm. Under low-angle and high-OA conditions, all specimens first exhibited elastic behavior, followed by a shift to viscoplastic behavior, which occurred before failure. OA ranging from 35° to 60° was associated with a brittle fracture mode in all specimens at E = 20 s, consistent with E = 10 s (Figure 5b). The load-bearing capacity advanced when exposure was prolonged (i.e., E = 30 s and 40 s), showing maximum displacement (>4.5 mm at 65° (E = 30 s) and >5 mm at 30° (E = 40 s)) and viscoelastic behavior before permanent deformation. Minimum displacement [<2 mm] was recorded at E = 30 s and 40 s, indicating brittle failure exhibiting elastic deformation prior to lasting distortion at 80° (Figure 5c,d). At E = 50 s, the load-displacement graph shows that the flexural strength declines and maximum specimens showed brittle failure [ε_fM_ = ε_fB_], as shown in Figure 5e. However, some specimens exhibited viscoelastic deformation at certain angles, which moved to plastic deformation with gradually increasing load.

Hence, the load-displacement graphs of the 3D-printed resins at various exposure times summarize the amount of elongation at break. The printed specimens’ bending moduli improved as the exposure period increased, because the three-point bending tests showed linear elastic deformation before the yield point. During the trial, none of the specimens broke, indicating they were extremely durable. Bending was reduced in E = 10 s compared to all printed examples. An increase in bending strength and good toughness was seen at E = 20 s, E = 30 s, and E = 40 s. However, the bending strength decreased, and fracture happened earlier at E = 50 s. Almost all the angles at which the load-displacement findings were examined showed similar patterns. With increasing load, a phenomenon at E = 10 s, 20 s, and 50 s showed rising modulus decreased flexural strength and elongation.

**Figure 6:**
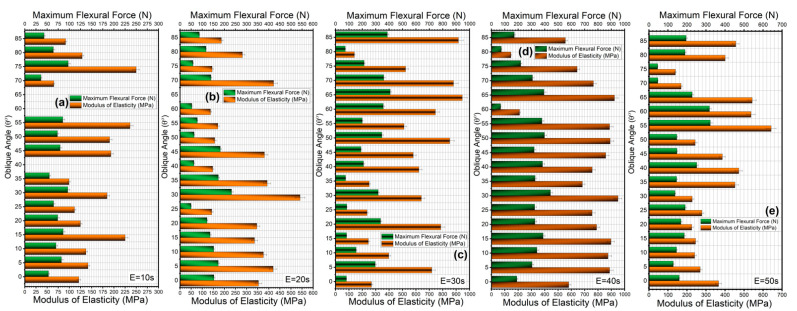
Moduli of elasticity and maximum flexural force results of fabricated rings at (**a**) E = 10 s, (**b**) E = 20 s, (**c**) E = 30 s, (**d**) E = 40 s, and (**e**) E = 50 s at different OA.

As shown in Figure 6, we also investigated the modulus of elasticity and the maximum applied flexural force prior to the permanent deformation of the PSR rings. At all exposure times, the produced specimens experienced an elastic deformation. A tiny amount of applied force generated significant elasticity before irreversibly deforming the specimen until it finally fractured as the applied force increased gradually over time.

### 3.5. Fracture Surface Analysis

Multiple fracture mechanisms are often found on a failed component, which can help investigators pinpoint the cause of the failure. In general, classification is based on the mechanism of fracture growth for the four types of fracture mechanisms: ductile failure, brittle fracture, intergranular fracture, or fatigue. The failure mechanism’s initial step is forming a free surface from an inclusion. The particle’s free surface creates a void. The strain and hydrostatic stress cause the void to expand. Finally, the voids expand to the point where they merge with or coalesce with other voids in the vicinity. As a result, a crack perpendicular to the tensile tension applied forms in the center of the voids. The applied stresses can alter the crack shape and configuration.

**Figure 7:**
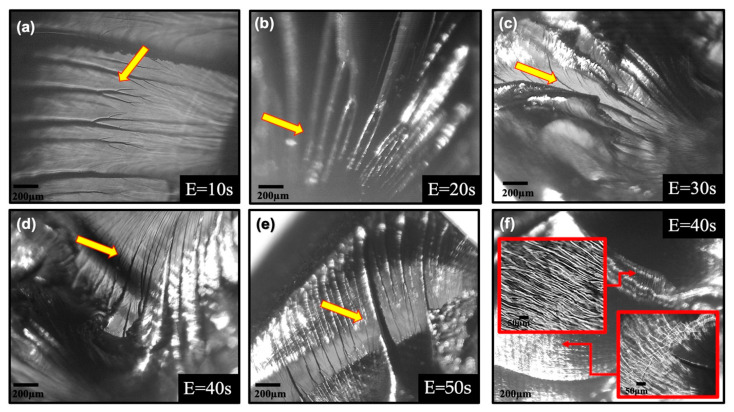
Fracture surface analysis of fabricated rings at (**a**) E = 10 s, (**b**) E = 20 s, (**c**) E = 30 s, (**d**) E = 40 s, (**e**) E = 50 s, (**f**) E = 40 s. Inset represents the enlarged view.

However, we observed ductile and brittle failure in our samples from the optical analysis of the fractured surface. Figure 7 displays fracture analysis of the surface displaying slow and unstable crack growth. The fracture grew at a very slow rate such that it could be seen with the naked eye after initiation at a low loading rate. When the rate of loading is high, cracks can grow in an unstable manner. The mechanism of fracture growth is brittle when it occurs during unstable growth progression, as shown in Figure 7a. The surface of the fracture was dull and fibrous. The surface of the fracture appeared to be smooth, and when viewed under high magnification, the elliptical marks that are indicative of brittle dynamic fracture could be observed. Besides this, we also saw the failing mechanism known as ductile failure, which is quite common. During ductile failure, the material is strained beyond its tensile strength. Before a ductile fracture collapses, the component undergoes significant plastic deformation on a macroscopic scale, as observed in Figure 7b–e. The cross-section is reduced or deformed as a result of failure. The eventual failure of the portion is indicated by the presence of shear lips at the end of the fracture as shown in Figure 7f. The change from sluggish crack propagation to rapid crack development is considered a transition stage. The speed of the crack immediately prior to transition increases as the rate of loading increases, reducing the degree of sluggish crack propagation. The loading rate and specimen configuration both play a role in determining when growth transitions from stable to unstable before the final fracture.

## 4. Conclusions

The results of this study demonstrate that the manufacturing mechanism significantly impacts the resilience of 3D-printed PSR rings. E = 30 s and 40 s were most appropriate for fabricating circular rings for various applications. The choice of a proper angle and exposure time is required to reduce mechanical failure to a minimum level, as well as enhancing the flexural strength in the fabricated specimen. The width gain is noticeable with increasing exposure time. It is possible to conclude that exposure and the oblique angle fabrication affect the flexural strength. The 3D-printed PSR load-displacement graphs highlight the degree of break elongation at various exposure times. Since three-point bending tests reveal linear elastic deformation before the yield point, increasing the exposure period enhanced the printed specimens’ bending modulus. This indicates that the test specimens were exceptionally resistant to breakage. E = 10s exhibited less bending than all other printed samples. At E = 20 s, E = 30 s, and E = 40 s, there was an increase in bending strength and good toughness. At E = 50 s, the bending strength dropped and fracture occurred sooner. Load-displacement patterns were consistent across most of the angles studied. At E = 10 s, 20 s, and 50 s, a rise in modulus, a fall in flexural strength, and elongation was observed. With increased exposure duration, PSR flexural strength and modulus rose. Due to the brittleness, after attaining specific peak values, both flexural strength and modulus diminished with increasing exposure duration. Thus, we conclude that E = 40 s is the best exposure time to fabricate the circular rings with increased flexural strength of 50 µm thickness.

## Figures and Tables

**Table 1:** 3D printing time of the ring specimens at a slice thickness of 50 μm and various slice irradiation times and angles.

Exposure Time	Time Completion Experiment [Hour (H)]																	
Angle (°)	0	5	10	15	20	25	30	35	40	45	50	55	60	65	70	75	80	85
10 s	1 H 37 Min						2 H 10 Min						3 H					
20 s	3 H 10 Min						4 H 10 Min						4 H 30 Min					
30 s	3 H 45 Min						4 H 30 Min						6 H					
40 s	5 H 30 Min						6 H 30 Min						7 H 25 Min					
50 s	5 H 40 Min						8 H 50 Min						10 H 30 Min					

## Data Availability

The data presented in this study are available on request from the corresponding author.

## Supplementary Materials

The following supporting information can be downloaded at: https://www.mdpi.com/article/10.3390/nano12152566/s1. Figure S1. Flexural strain at break (i-εfB) and maximum flexural strain (ii-εfM) graphs of fabricated rings at (a) E = 10 s, (b) E = 20 s, (c) E = 30 s, (d) E = 40 s, and (e) E = 50 s at different OA.
