# Supplementary material for: Influence of Exposure Period and Angle Alteration on the Flexural Resilience and Mechanical Attributes of Photosensitive Resin
Source: Nanomaterials (Basel). 2022 Jul 26;12(15):2566. doi: 10.3390/nano12152566 (PMC9332362; doi:10.3390/nano12152566)
Supplement: Supplementary file 1 [file nanomaterials-12-02566-s001.zip › nanomaterials-1770968-supplementary.pdf]

nanomaterials

MDPI

Supporting Information

Article

Influence of Exposure Period and Angle Alteration on the Flexural Resilience and Mechanical Attributes of Photosensitive Resin

Sadaf Bashir Khan [1,2,3,4], Nan Li [1,4], Jiahua Liang [4], Chuang Xiao [1,4], Xiaohong Sun [3,*] and Shenggui Chen [1,2,4,*]

[1] Dongguan University of Technology, Dongguan 523808, China; sadafbashirkhan@dgut.edu.cn (S.B.K.); dglgln@163.com (N.L.); xiaoxcuang@163.com (C.X.)
[2] School of Art and Design, Guangzhou Panyu Polytechnic, Guangzhou 511483, China
[3] School of Materials Science and Engineering, Tianjin University, Tianjin 300350, China
[4] Dongguan Institute of Science and Technology Innovation, Dongguan University of Technology, Dongguan 523808, China; jiahua_liang1114@163.com (J.L)
* Correspondence: sunxh@tju.edu.cn (X.S.); dgutchensg@163.com (S.C.)

Supporting Information

- 3D-printed PSR strain (ε_{fB} & ε_{fM}) Analysis
- Figure S1

3D Printed PSR Strain (ε_{fB} & ε_{fM}) Analysis

Figure S1 represents the flexural strain ε_{fB} displaying the strain specimens underwent before breaking and flexural strain ε_{fM}: the specimens' behavior at maximum flexural stress ε_{fM} at various oblique angles. At E = 10s and fabrication angles 0°, 15°, and 75°, the specimens experienced maximum flexural strain before permanent deformation and ε_{fM}= ε_{fB}. The minimum flexural strain was observed at OA 70°. Specimens underwent almost similar ε_{fM}= ε_{fB} at all the OA, as clearly identified in the flexural strain graphs displayed in Figure S1. At E = 20s, the rings fabricated at mid-OA (40°, 50°, 55°, 60°, 65°), HA（75°), and LA (25°) specimens underwent brittle failure before permanent deformation, having ε_{fM}= ε_{fB} with the lowest strain of 3.6% at angles 25° and 60°. At all other OA, the rings underwent viscoelastic deformation with varying ε_{fM} and ε_{fB} (Figure S1b). At E = 30s and 40 s, the minimum ε_{fM} =ε_{fB} >1.7~1.05% was observed at OA=80°. The rings underwent viscoelastic deformation with varying ε_{fM} and ε_{fB} at other angles. Specimens with brittle failures had ε_{fM}= ε_{fB}. All other specimens showing viscoelastic deformation or viscoplastic failures had varying flexural strain at break (Figure S1 (c and d)). In the case of E = 50 s, again, the rings underwent maximum flexural strain of 8.8% before going to permanent deformation at 55°: ε_{fM} =7.18%.

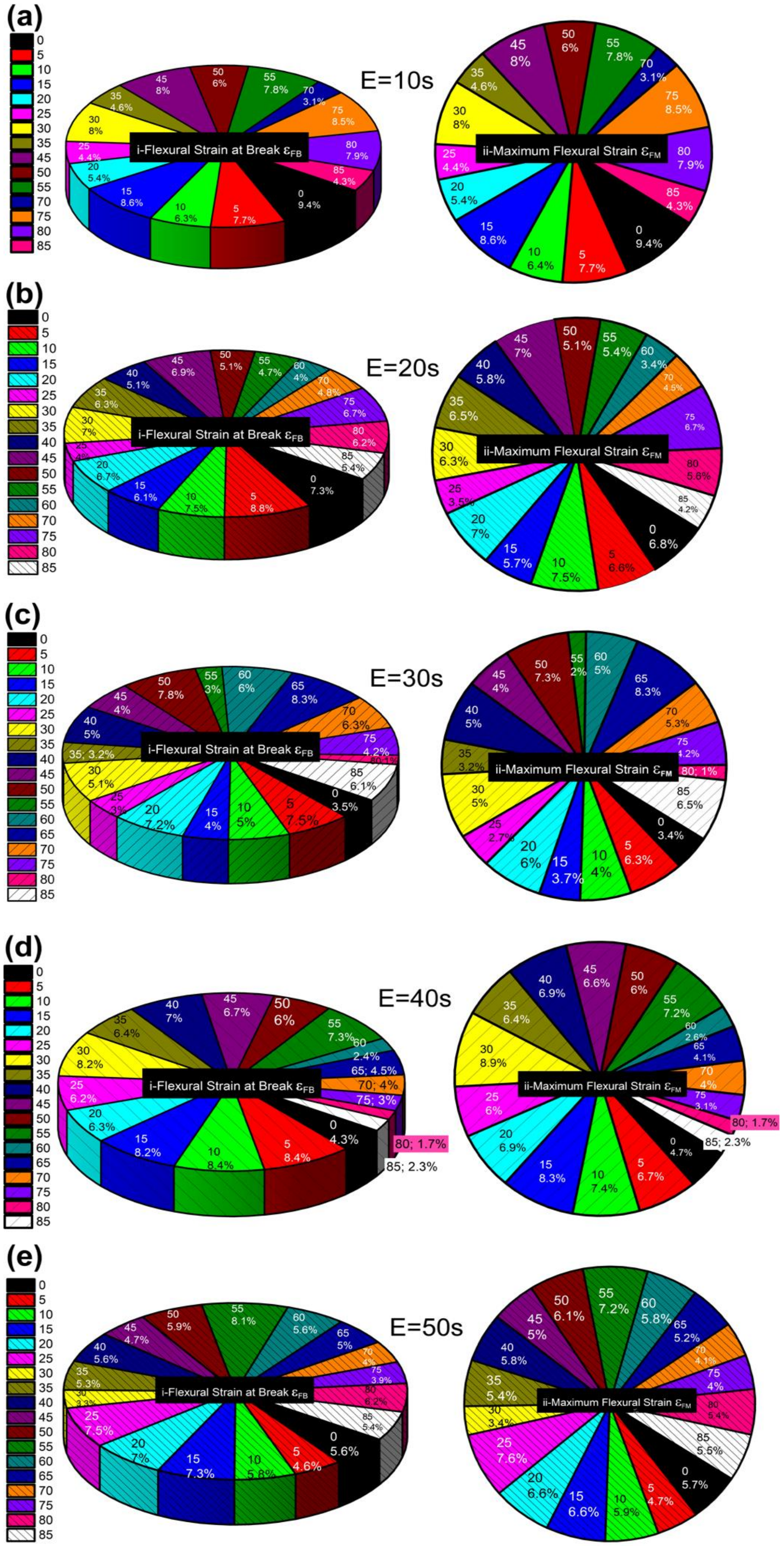

Figure S1. Flexural strain at break (i- ε_{fB}) and maximum flexural strain (ii- ε_{fM}) graphs of fabricated rings at (**a**) E = 10 s, (**b**) E = 20 s, (**c**) E = 30 s, (**d**) E = 40 s, and (**e**) E = 50s at different OA.
